# Stress hormone signalling inhibits Th1 polarization in a CD4 T‐cell‐intrinsic manner via mTORC1 and the circadian gene *PER1*

**DOI:** 10.1111/imm.13448

**Published:** 2022-03-02

**Authors:** Christophe M. Capelle, Anna Chen, Ni Zeng, Alexandre Baron, Kamil Grzyb, Thais Arns, Alexander Skupin, Markus Ollert, Feng Q. Hefeng

**Affiliations:** ^1^ Department of Infection and Immunity Luxembourg Institute of Health (LIH) Esch‐sur‐Alzette Luxembourg; ^2^ Faculty of Science Technology and Medicine University of Luxembourg Esch‐sur‐Alzette Luxembourg; ^3^ Luxembourg Centre for Systems Biomedicine (LCSB) University of Luxembourg Belvaux Luxembourg; ^4^ Department of Dermatology and Allergy Center Odense Research Center for Anaphylaxis (ORCA) University of Southern Denmark Odense Denmark; ^5^ Institute of Medical Microbiology University Hospital Essen University of Duisburg‐Essen Essen Germany

**Keywords:** adrenergic signalling, circadian rhythm, neuroimmunology, PER1, stress, T‐cell differentiation

## Abstract

Stress hormones are believed to skew the CD4 T‐cell differentiation towards a Th2 response via a T‐cell‐extrinsic mechanism. Using isolated primary human naïve and memory CD4 T cells, here we show that both adrenergic‐ and glucocorticoid‐mediated stress signalling pathways play a CD4 naïve T‐cell‐intrinsic role in regulating the Th1/Th2 differentiation balance. Both stress hormones reduced the Th1 programme and cytokine production by inhibiting mTORC1 signalling via two parallel mechanisms. Stress hormone signalling inhibited mTORC1 in naïve CD4 T cells (1) by affecting the PI3K/AKT pathway and (2) by regulating the expression of the circadian rhythm gene, period circadian regulator 1 (*PER1*). Both stress hormones induced the expression of *PER1*, which inhibited mTORC1 signalling, thus reducing Th1 differentiation. This previously unrecognized cell‐autonomous mechanism connects stress hormone signalling with CD4 T‐cell differentiation via mTORC1 and a specific circadian clock gene, namely *PER1*.

## Graphical abstract

Using primary human naïve and memory CD4 T cells, here we show that both adrenergic‐ and glucocorticoid‐mediated stress signalling pathways play a CD4 naïve T‐cell‐intrinsic role in regulating the Th1/Th2 differentiation balance. Both stress hormones induced the expression of period circadian regulator 1 (PER1), which inhibited mTORC1 signalling, thus reducing Th1 differentiation.

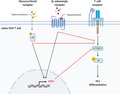

## INTRODUCTION

During a stress response, the neuroimmune interaction is mediated by the sympathetic nervous system and the adrenal gland releasing particular stress hormones in the vicinity of immune cells expressing the corresponding receptors, such as the adrenergic receptors (e.g. β2 adrenergic receptor, β_2_AR) and glucocorticoid receptor (GR). The stress hormones, for example (nor)adrenaline and glucocorticoids, were initially considered purely immunosuppressive and generally harmful [1], especially in autoimmune, inflammatory or infectious diseases [2, 3, 4, 5, 6] and cancer [7, 8, 9], among others [10]. In the meantime, the levels of various stress hormones are oscillating according to a diurnal cycle and are strictly regulated by the circadian rhythm regulatory machinery [11, 12, 13]. The circadian ‘clock’ machinery and its components play a vital role in regulating various immune functions, including trafficking [14, 15, 16], Th2 and Th17 differentiation [17, 18]. The circadian machinery has been found to be dysregulated in many complex diseases, including various immune‐associated diseases [19, 20, 21, 22, 23]. However, possibly due to the dynamic ‘timing’ complexity underlying the circadian rhythm, it hitherto largely remains elusive whether and how the potential bilateral interactions between stress signalling and circadian regulatory genes mediate different effector or regulatory functions of various immune subsets.

Following extensive studies, stress hormones have exhibited many types of effects in various immune cell types, either promoting or reducing specific immune functions in different health and disease contexts [24]. For instance, total CD4 T cells reduce the expression of cytokines, such as IFN‐γ and IL‐2 [25, 26] upon stress hormone signalling by a multitude of pathways involving cAMP/PKA [27], NF‐κB [28] and by inhibiting early TCR singling [29], among others [30]. On the contrary, a specific subset of CD4 T cells, regulatory CD4 T cells (Treg), benefit from stress hormone signalling by increasing their suppressive capability [31, 32, 33]. Furthermore, activation of β_2_AR was recently shown to skew CD4 differentiation towards Tregs [34]. Activation of β_2_AR can also augment IL‐17 expression in another purified T‐helper subset, the Th17 cells, following stimulation [35]. As for myeloid cells, dendritic cells (DCs), monocytes and macrophages have been shown to reduce the expression of pro‐inflammatory cytokines, such as TNF‐α and IL‐12, but increase the expression of anti‐inflammatory cytokines, such as IL‐10 and IL‐4 [36, 37, 38, 39]. Stress hormones also affect various other types of immune cells, such as B cells [40, 41, 42], CD8 T cells [43] and NK cells [44], among others (reviewed here [45, 46]). In the meantime, treatment with a β_2_AR agonist in TCR‐stimulated human peripheral blood mononuclear cells also inhibits the secretion of the Th1 cytokine IFN‐γ while enhancing IL‐17 in culture media [35]. The complexity of the stress hormone‐mediated effects on immune cells is further increased by the potential expression of multiple (sub)types of adrenergic receptors, as targeting alpha‐ or beta‐AR could generate differential outcomes, as shown in the disease model of adjuvant arthritis [47].

Connecting the dots between the various aforementioned effects on different immune cells, the stress hormones might more specifically inhibit cellular immunity and Th1 responses, while favouring a Th2 response and humoral immunity [48, 49, 50]. In the existing paradigm, this Th1 programme inhibiting process is believed to be regulated via a T‐cell extrinsic manner, that is, by suppressing the production of IL‐12 in DCs, as demonstrated in both human and murine cells [36, 37, 39]. Since IL‐12 is critical for initiating Th1 differentiation [51, 52], the secretion of stress hormones diminishes Th1 differentiation indirectly via DCs. Therefore, a stress response favours Th2 responses, which can explain stress‐induced exacerbation of allergic diseases [53], characterized by a dominant Th2 pathology. At the same time, the stress‐induced decrease in Th1 immunity has the functional consequence of increasing susceptibility to viral infections [4, 5] and reducing anti‐tumour immunity [7, 54]. Indeed, blocking the adrenergic or glucocorticoid signalling increased anti‐tumour immune responses and treatment efficacy [55, 56]. Although T‐cell‐extrinsic mechanisms under physiological and pathological conditions have been described as aforementioned, it still remains unknown whether stress hormones engage a CD4 T‐cell‐intrinsic manner to regulate T‐cell differentiation.

Using sorted primary human CD4 naïve and memory T cells, we show that stress hormone signalling in naïve CD4 T cells intrinsically inhibited Th1 polarization via the gene period circadian regulator 1 (*PER1*) and the mTORC1 signalling pathway.

## MATERIALS AND METHODS

### Primary naïve and memory CD4 T‐cell isolation

Buffy coats from more than 70 healthy donors were generously provided by the Red Cross Luxembourg. Due to the sequential design of the project, the cells from different donors were used to perform different experiments, although always with a certain overlap. Here, the overlap essentially referred to the experimental readouts between two relevant experiments to control the operational technical correctness. For instance, when measuring readouts related to cell signalling, such as the mTOR pathway, the Th1/Th2 phenotype was also measured as a control to ensure that the signalling was related to the observed differentiation phenotype. We first isolated total CD4 cells by adding the RosetteSep™ Human CD4+ T cell Enrichment Cocktail (15062, StemCell) to undiluted blood at a concentration of 50 µl/ml and incubated the mix for 30 min at 4°C. Next, the same volume of FCM buffer (Ca^2+^/Mg^2+^ free PBS +2% FBS) was added to the blood and carefully transferred to a SepMate™ 50 tubes (85450, StemCell), on top of the Lymphoprep solution (07801, StemCell) in order to isolate the total CD4+ T cells by gradient centrifugation at 1200×g for 20 min. The cells were washed three times in FCM buffer and stained for FACS sorting. Total CD4+ T cells were stained (Table S1) with mouse monoclonal [RPA‐T4] anti‐human CD4 FITC (555346, BD) (dilution 1:20), mouse monoclonal [M‐A251] anti‐human CD25 APC (555434, BD) (dilution 1:20), mouse anti‐human CD45RA [HI100] Pacific Blue (BioLegend, 304118), mouse anti‐human CD45RO [UCHL1] PE‐CF594 (BD, 562299) and LIVE/DEAD^®^ Fixable Near‐IR Dead Cell Stain (L10119, Thermo Fisher Scientific) (dilution 1:500). Primary naïve (CD4^+^CD25^low^CD45RA^+^) and memory (CD4^+^CD25^low^CD45RO^+^) CD4 T cells were isolated on a BD FACSAria^TM^ III cell sorter (BD Biosciences).

Alternatively, naïve/memory CD4 T cells were isolated with the EasySep™ Human Naïve CD4+ T Cell Isolation Kit (StemCell, #19555) or the EasySep™ Human Memory CD4+ T Cell Enrichment Kit (StemCell, #19157), following the manufacturer's instructions.

For some other donors, PBMCs were first isolated by gradient centrifugation and naïve/memory CD4 T cells were isolated with the EasySep or Naïve CD4+ T Cell Isolation Kit II, human (Miltenyi Biotec, 130‐094‐131) and Memory CD4+ T Cell Isolation Kit, human (Miltenyi Biotec, 130‐091‐893), following the manufacturer's instructions.

The results were consistent no matter which isolation method was used; however, the highest purity of cells was obtained through FACS sorting (>99%, Figure S1A).

### Culture conditions and treatment of primary T cells

Sorted naïve and memory CD4+ T cells were cultured in IMDM (21980‐032, Thermo Fisher Scientific) complete medium, supplemented with 10% heat‐inactivated (56°C, 45 min) fetal bovine serum (FBS) (10500‐064, Thermo Fisher Scientific), 1× penicillin + streptomycin (15070‐063, Thermo Fisher Scientific), 1× MEM non‐essential amino acids (M7145, Sigma‐Aldrich) and 50 μM β‐mercaptoethanol (21985‐023, Thermo Fisher Scientific) for 48–72 h before every experiment. This is to ensure that there are no more circadian fluctuations in the cells as the CD4 T‐cell‐intrinsic fluctuations of the circadian genes were shown to be abolished after 48 h of cell culture [57].

Between 5 × 10^5^ and 1 × 10^6^ naïve or memory CD4 T cells were seeded in 1 ml of culture media in 48‐well plates in the presence or absence of different compounds: isoproterenol hydrochloride (ISO), 50 μM (Sigma‐Aldrich, I6504); forskolin (Forsk), 5 μM (Sigma‐Aldrich, F3917); hydrocortisone (HC), 0.5 μM (Sigma‐Aldrich, H0396); rapamycin, 5 nM (StemCell, 73362) and L‐(‐)‐noradrenaline (+)‐bitartrate salt monohydrate (norepinephrine, NE, Sigma‐Aldrich, A9512; NE was only used in Figure S1D). Of note, although the concentrations mentioned above were used in most of the experiments, different concentrations might have been used in a few experiments for different purposes and were then specified in the corresponding figures.

The compounds were added 1 h prior to TCR stimulation by soluble anti‐CD3/anti‐CD28 antibodies (25 µl/ml ImmunoCult™ Human CD3/CD28 T Cell Activator) (10971, StemCell) and incubated for different durations following stimulation depending on the experiment. For most of the flow cytometry staining following the treatment with different compounds, naïve and memory CD4 T cells were stimulated for 48 h unless otherwise specified.

### siRNA knockdown in primary T cells

Targeted gene expression (*PER1*, *PER2*, *PER3*) was knocked down as described elsewhere [58], in up to 5 × 10^6^ cells using the P3 Primary Cell 4D‐Nucleofector X Kit L (V4XP‐3024, Lonza) with 90 µl P3 primary cell solution and 100 pmol of corresponding si_RNA (resuspended in 10 ul RNAse‐free H2O): si_Non‐Specific scrambled control siRNA (si_NS or si_CTRL) (SC‐37007, Santa Cruz), si_*PER1* (SI00040537, Qiagen), si_*PER2* (SI02632189, Qiagen) and si_*PER3* (SI00117530, Qiagen). siRNA transfection was done by using the Amaxa 4D‐Nucleofector™ X System (Lonza) following the manufacturer's recommended programme for primary human T cells (with the programme code EO‐115). Following transfection, the naïve or memory CD4 T cells were transferred into a 12‐well plate with pre‐warmed complete IMDM and incubated at 37°C for 24 h. The next day, the cells were stimulated with 25 µl/ml of soluble antibodies (ImmunoCult™ Human CD3/CD28 T Cell Activator) (10971, StemCell) for 24 h in 1 ml in a 48‐well plate. The knockdown efficiency was assessed by quantitative real‐time PCR (qPCR) to ensure siRNA‐induced reduction in the targeted gene.

### Flow cytometry analysis

Naïve and memory CD4 T cells were harvested by centrifugation (250×g, 10 min) at the end of the experiment, washed once in FCM buffer (Ca^2+^/Mg^2+^ free PBS +2% FBS) and resuspended in the staining mastermix for the surface staining. Antibodies used are listed in Table S2. Of note, not necessarily all the abs listed there were used in the same staining panel. Following the surface staining, the cells were washed three times in FCM buffer and fixed for 1h at room temperature (RT) using the fixation buffer of the True‐Nuclear Transcription Factor Buffer Set (BioLegend, 424401). After fixation, the cells were washed once in permeabilization (Perm) buffer and resuspended in Perm buffer, containing the antibodies for the intracellular staining (listed in 2nd half of Table S2), and incubated for 30 min at RT. The cells were washed 3 times in Perm buffer and then resuspended in FCM buffer for the acquisition on the BD LSRFortessa^TM^.

### Intracellular cytokine staining

4 h before harvesting the cells, GolgiStop (BD, 554724) was added to the cell cultures to inhibit the secretion of the cytokines, leading to an accumulation inside the cells. Naïve and memory CD4 T cells were harvested by centrifugation (250×g, 10 min) at the end of the experiment, washed once in FCM buffer (Ca^2+^/Mg^2+^ free PBS +2% FBS) and resuspended in the staining master mix for the surface staining (CD4 FITC and L/D APC‐Cy7). Following the surface staining, the cells were washed three times in FCM buffer and fixed for 30 min at 4°C using the Cytofix/Cytoperm buffer set (BD, 554714). After fixation, the cells were washed once in Perm/Wash buffer and resuspended in Perm/wash buffer, containing the antibodies for the intracellular staining (refer to Table S3), and incubated for 30 min at RT. The cells were washed three times in Perm/Wash buffer and resuspended in FCM buffer for the acquisition on the BD LSRFortessa^TM^.

### Supernatant cytokine measurement by MSD assay

The supernatant of the naïve and memory CD4 cell cultures was collected after 24 h or 48 h (depending on the experiment) following stimulation by centrifuging down the cells (250×g, 10 min). The concentration of a selection of CD4 cytokines, including Th1, Th2 and other cytokines (IFN‐γ, TNF‐α, GM‐CSF, IL‐2, IL‐4, IL‐5, IL‐13, IL‐17A, IL‐17A/F, IL‐21, IL‐9 and IL‐10), was measured in undiluted culture medium using the MSD U‐PLEX Human Biomarker Group 1 Kit (MSD, K15067L‐1) according to the manufacturer's instructions. The plates were read by the MESO QuickPlex SQ 120 instrument, and the data were analysed with the provided MSD DISCOVERY WORKBENCH software.

### RNA extraction, cDNA synthesis and qPCR

The RNeasy Mini Kit (74106, Qiagen) or RNeasy Micro Kit (74004, Qiagen) was used for RNA extraction according to the manufacturer's instructions and including a genomic DNA digestion step with DNAse I (79254, Qiagen). The cells were lysed in RLT buffer (79216, Qiagen), supplemented with 1% beta‐mercaptoethanol (63689, Sigma‐Aldrich) and frozen at −20°C for several hours or days until the RNA extraction. The NanoDrop 2000c Spectrophotometer (Thermo Fisher Scientific) was used to measure RNA concentration.

For the cDNA synthesis, we followed a very similar procedure described elsewhere [59]. To ease the comprehension of this work, we described the procedures here again. The SuperScript™ IV First Strand Synthesis System (18091050, Thermo Fisher Scientific) was used, with a maximum input of 500 ng of RNA. The master mix for the first step included per sample: 0.5 µl of 50 µM Oligo(dT)20 primers (18418020, Thermo Fisher Scientific), 0.5 µl of 0.09 U/µl Random Primers (48190011, Thermo Fisher Scientific), 1 µl of 10 mM dNTP mix (18427013, Thermo Fisher Scientific) and RNAse‐free water for a final volume of 13 µl. The C1000 Touch Thermal Cycler (Bio‐Rad) or UNO96 HPL Thermal Cycler (VWR) were used for both steps. For the first step, the following programme was used: 5 min at 65°C, then 2 min at 4°C. Before the second reaction step, the mix was supplemented with 40 U RNaseOUT™ Recombinant Ribonuclease Inhibitor (10777019, Thermo Fisher Scientific), 200 U SuperScript™ IV Reverse Transcriptase (18090050, Thermo Fisher Scientific), a final concentration of 5 mM dithiothreitol (DTT) (707265ML, Thermo Fisher Scientific) and 1x SuperScript^TM^ IV buffer for a final reaction volume of 20 µl. For the second step, the following programme was used: 10 min at 50°C, 10 min at 80°C and 4°C until the samples were picked up. The obtained cDNA was diluted 3 times with nuclease‐free water to a final volume of 60 µl.

For the qPCR, a master mix for the following reaction mixture was prepared per well: 5 µl of the LightCycler 480 SYBR Green I Master Mix (04707516001, Roche), 2.5 µl cDNA and 2.5 µl primers in a total reaction volume of 10 μl. The PCR was performed in a CFX384 Touch Real‐Time PCR System (Bio‐Rad), using LightCycler 480 Multiwell 384‐well plates (04729749001, Roche) sealed with the LC 480 Sealing Foil (04729757001, Roche). The following programme was used: 5 min at 95°C; 45 cycles of 10 s at 55°C, 20 s at 72°C and 10 s at 95°C; and melting curve at 65–97°C. The results were analysed using the 2^−ΔΔCt^ method. Primers used for qPCR were as follows: RPS9 (QT00233989, Qiagen) as a reference gene, *PER1* (QT00069265, Qiagen), *PER2* (QT00011207, Qiagen), *PER3* (QT00097713, Qiagen), *ARNTL1* (BMAL1) (QT00011844, Qiagen), *CLOCK* (QT00054481, Qiagen), *CRY1* (QT00025067, Qiagen), *CRY2* (QT00094920, Qiagen), *NFIL3*, also known as E4BP4 (QT00013944, Qiagen), *NR1D1* (REV‐ERBa) (QT00000413, Qiagen), *IL2* (QT00015435, Qiagen), *IFNG* (QT00000525, Qiagen) and *TBX21* (T‐bet) (QT00042217, Qiagen).

### ATP measurement

The CellTiter‐Glo^®^ Luminescent Cell Viability Assay (G7570, Promega) was used to measure the ATP concentration in the cells. 2 × 10^5^ cells were lysed and prepared according to the manufacturer's recommendations.

### cAMP assay

The intracellular cAMP concentration following the treatment with different compounds was analysed in undiluted samples with the cAMP 96‐well kit (MSD, K150W5D), following the manufacturer's protocol and measured by the MESO QuickPlex SQ 120 instrument.

### Statistical analysis

Statistical analysis was performed in GraphPad Prism 9.0 using either a one‐way ANOVA with Dunnett's multiple comparison correction or a paired two‐tailed t‐test, depending on the features of the corresponding experiment. The test used for the different figures was specified in the figure legends. The error bars in the related types of figures represent the standard deviation (SD).

## RESULTS

### Stress hormones decrease the activity and proliferation of naïve and memory CD4 T cells

The general immunosuppressive role of stress hormones has been established for several decades. In the meantime, there is a substantial interaction between stress reactions and the circadian rhythm, that is our body internal 24‐h oscillating clock [60], in various immune responses. However, it still remains elusive whether there is a differential effect of stress hormones in specific subsets of CD4 T cells. Furthermore, the potential critical interactions between stress signalling and circadian rhythm have not yet been investigated in this context. Therefore, we aimed to illustrate this particular mechanistic aspect in this work.

**FIGURE 1:**
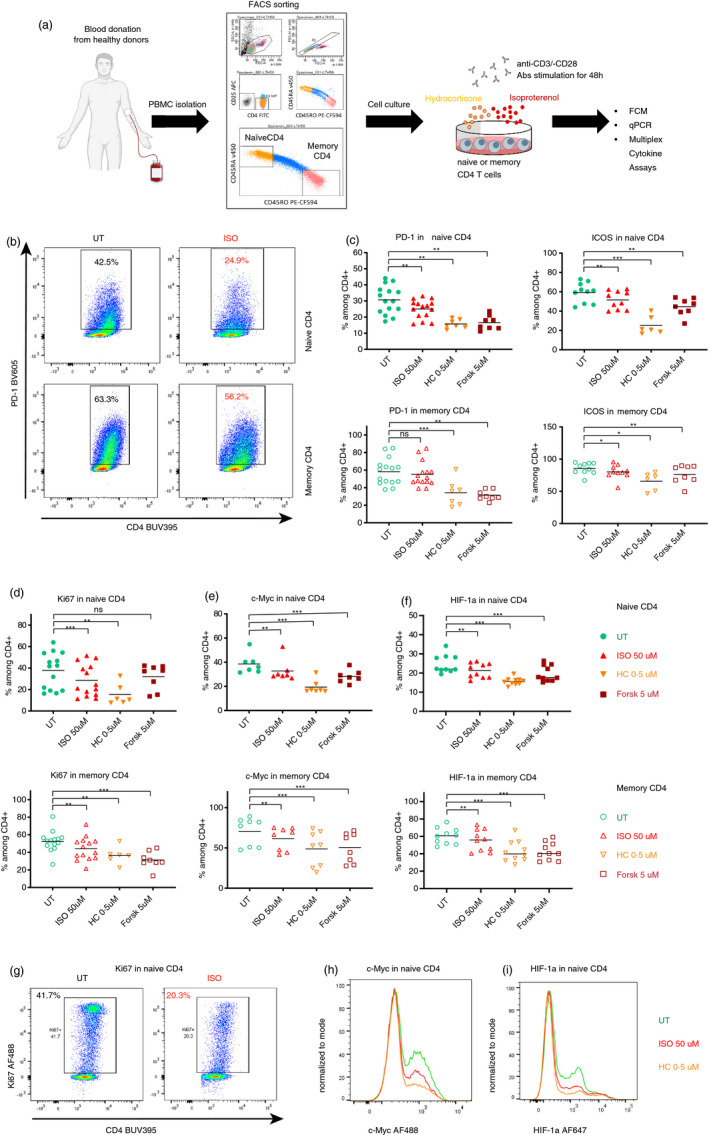
Stress hormones decrease the activity and proliferation of naïve and memory CD4 T cells. (a) Graphical representation of the experimental set‐up. Naïve (CD4^+^CD25^low^CD45RA^+^) and memory (CD4^+^CD25^low^CD45RO^+^) CD4 T cells were isolated by gradient centrifugation and FACS sorting. The isolated cells were exposed to stress hormone analogues isoproterenol (ISO, β_2_AR agonist) or hydrocortisone (HC, synthetic glucocorticoid) or forskolin (Forsk) for 1 h prior to TCR stimulation and were harvested at different time‐points following stimulation for different analyses. (b) Representative flow cytometry plots showing the decreased expression of PD‐1 after ISO treatment, following 48‐h TCR stimulation. (c) Scatter dot plots showing the effect of ISO, HC and forskolin (Forsk) on the expression of PD‐1 (*n* = 7–15) and ICOS (*n* = 6–10) in naïve and memory CD4, measured by flow cytometry. (d–f) Scatter dot plots showing the effect of ISO, HC and forskolin (Forsk) on the expression of Ki67 (*n* = 6–14) (d), c‐Myc (*n* = 8) (e) and HIF‐1α (*n* = 10) (f) in naïve and memory CD4, measured by flow cytometry. (g) Representative flow cytometry plots showing the decreased expression of Ki67 after ISO treatment. (h, i) Representative histogram overlay showing the expression of c‐Myc (h) and HIF‐1a (i) among living CD4 T cells in the absence or presence of ISO or HC. FACS, fluorescence‐activated cell sorting; FCM, flow cytometry analysis; qPCR, quantitative PCR; UT, untreated but still stimulated; US, unstimulated. Each individual value in the scatter dot plots was displayed in each group. The horizontal bars in (c‐f) represent the mean. The results in (c‐f) were analysed using one‐way ANOVA with multiple comparison correction. ns, not significant; *p ≤ 0.05, **p ≤ 0.01 and ***p ≤ 0.001. See also Figure S1

We first sought to investigate the suppressive effect of stress hormones on general activation status of naïve (Tn) and memory (Tm) CD4 T cells following TCR stimulation. To this end, we sorted CD4 Tn and Tm cells from peripheral blood mononuclear cells (PBMCs) of healthy human donors (Figure S1A). We exposed them for 1 h to isoproterenol (β_2_AR agonist) or hydrocortisone (HC) (synthetic glucocorticoid) before a 48‐h anti‐CD3/‐CD28 stimulation and assessed the levels of different activation and proliferation markers by flow cytometry (Figure 1a). Confirming the known immunosuppressive role of the stress hormones, we observed a decrease in the expression of the activation markers PD‐1 and ICOS, and the proliferation marker Ki67 in CD4 Tn (Figure 1b–d). In CD4 Tm, this effect was less pronounced and only significant for ICOS and Ki67 (Figure 1b–d). In line with the reduced activation and proliferation, both CD4 Tn and Tm treated with stress hormone analogues showed a decreased expression of cMyc and HiF1α (Figure 1e–i). These results indicate a reduced metabolic activity, as both signalling molecules are crucial for the glycolytic switch upon T‐cell activation [61, 62]. Indeed, ISO and HC reduced expression of GLUT1 and ATP production in CD4 Tn and Tm (Figure S1B, C), indicating an abridged level of glucose uptake and a lower metabolic output. These data are in line with a recent report showing that adrenergic signalling blocks the metabolic reprogramming by inhibiting glucose uptake, although in CD8 T cells [43]. Adrenergic signalling downstream of the β_2_AR activates the adenylate cyclase to produce cAMP, which then acts as a second‐messenger on downstream targets. Forskolin (Forsk), an adenylate cyclase agonist, leads to high levels of cAMP independent of β_2_AR signalling and was used as a positive control for cAMP activity in our study (Figure S1d). When exposed to Forsk, the markers reflecting T‐cell activation (PD‐1, ICOS), proliferation (Ki67) and metabolic activity (reflected by the readouts of ATP, GLUT1, cMyc and HIF1a) were decreased in both CD4 Tn and Tm (Figures 1b–f and S1B, C). This indicates the involvement of previously described cAMP‐dependent mechanisms of ISO, downstream of β_2_AR signalling to suppress CD4 T cells [27]. Thus, we successfully showed that both CD4 Tn and Tm displayed decreased activation, proliferation and metabolic activity when treated with a β_2_AR agonist or a synthetic glucocorticoid, even though CD4 Tm seemed to be less sensitive to these treatments.

### Stress hormones shift the balance of the T‐helper differentiation in a naïve CD4 T‐cell‐intrinsic manner

Many studies have demonstrated that stress responses favour a Th2 over a Th1 differentiation [48] in a T‐cell‐extrinsic manner, that is indirectly by inhibiting the production of DC‐derived IL‐12 [36, 37]. However, such a T‐cell‐extrinsic mechanism cannot rule out a possible CD4 T‐cell‐intrinsic role of stress hormones on T‐cell differentiation. Therefore, we studied the possible existence of a naïve CD4 T‐cell‐intrinsic mechanism, independent of exogenous cytokines, to regulate Th cell polarization. To investigate this, we exposed sorted CD4 Tn and Tm to ISO, HC or Forsk and measured the expression of the lineage transcription factors (LTFs) of different T‐helper subsets 48 h after TCR stimulation. As we were only interested in the potential existance of a T‐cell‐intrinsic mechanism in this work, we did not add any Th1‐, Th2‐ or Th17‐polarizing cytokine cocktails to the media. Similarly, we also did not co‐culture CD4 Tn with antigen‐presenting cells as the T‐cell‐extrinsic mechanism has been well demonstrated and validated by several other groups in both human and murine cells [36, 37, 39].

**FIGURE 2:**
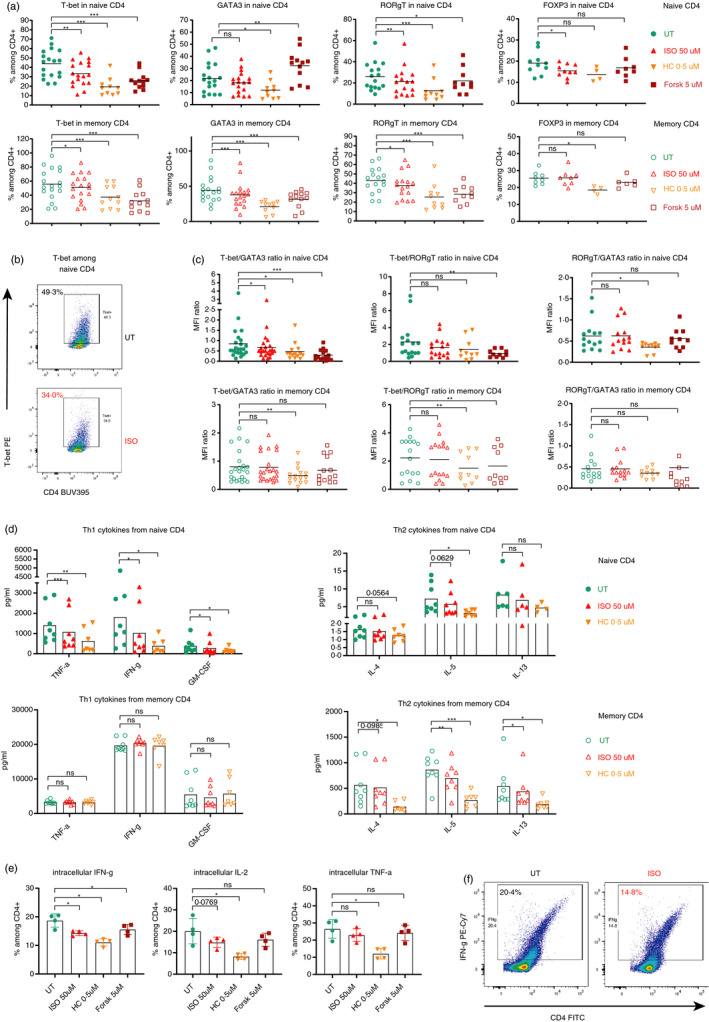
Stress hormones intrinsically shift the balance of the T‐helper programmes in naïve CD4 T cells. (a) Expression of the CD4 master/lineage transcription factors for the Th1 (T‐bet) (*n* = 10–18), Th2 (GATA3) (*n* = 01–18), Th17 (RORγT) (*n* = 10–16) and Treg (FOXP3) (*n* = 4–10) cells in naïve (top) and memory (bottom) CD4 T cells after 48h TCR stimulation in the absence or presence of different stress hormone analogues, measured by flow cytometry. (b) Representative flow cytometry plots for the expression of T‐bet and CD4 among naïve CD4 T cells in the absence or presence of ISO. (c) Ratios between the geometric mean (geomean or MFI) of different transcriptions factors in naïve (top) and memory (bottom) CD4 T cells. (d) Secreted cytokines, measured in the culture medium after 48 h of TCR stimulation using the MSD multiplex cytokine assays. Cytokines secreted by naïve CD4 T cells are shown in the top row, and memory CD4 in the bottom row. Th1 cytokines (left) and Th2 cytokines (right) are grouped in different graphs (*n* = 7–8). (e) Graphs showing the levels of intracellular cytokines in naïve CD4 after ISO, HC or Forsk treatment and 48‐h TCR stimulations. (f) Representative flow cytometry plots for the expression of IFN‐g and CD4. Isoproterenol (β_2_AR agonist); hydrocortisone (HC) (synthetic glucocorticoid); UT, untreated but still stimulated; US, unstimulated. Each individual value in each group in the scatter dot plots is displayed. The horizontal bars in (a, b) and the boxes in (c) represent the mean. Data in e presented as mean ± standard deviation (SD). The results in (a–e) were analysed using one‐way ANOVA with multiple comparison correction. ns, not significant; *p ≤ 0.05, **p ≤ 0.01 and ***p ≤ 0.001. See also Figures S2 and S3

In our experiments with purified CD4 subsets, both ISO and HC selectively decreased the expression of the Th1 and Th17 LTFs, T‐bet and RORγT (in a dose‐dependent manner for T‐bet; Figure S2A), respectively, whereas the Th2 LTF GATA3 remained unchanged in CD4 Tn (Figure 2a, b). In contrast, CD4 Tm displayed a more universal decrease in the LTFs, although GATA3 was most significantly decreased (Figure 2a). In addition, we only observed a slight decrease in FOXP3 expression in CD4 Tn following ISO and in CD4 Tm following HC (Figure 2a). Overall, these data indicate a cell‐type‐specific effect of the stress hormone analogues on CD4 Tn or Tm, where Th1 and Th17 polarization was most reduced in CD4 Tn, whereas Tm showed the most significant decrease in the expression of the Th2 LTF. In fact, it is the balance between those LTFs, instead of their absolute expression levels, that determines the functional outcome of the CD4 T‐cell differentiation. Therefore, we calculated the ratios between various LTFs to analyse which of the signals is relatively dominant. We found that only the T‐bet/GATA3 ratio, reflecting the Th1/Th2 balance, was consistently reduced in CD4 Tn in an ISO dose‐dependent manner (Figures 2c and S2A), but not in CD4 Tm (Figures 2c and S2A). When treated with HC, both CD4 Tn and Tm showed a decreased Th1/Th2 ratio, indicating that β_2_AR has a more differential effect than HC. Interestingly, Forsk treatment even increased GATA3 expression, while still decreasing the expression of T‐bet and RORγT in CD4 Tn (Figure 2a), further pushing towards a Th2 polarization (Figure 2c).

Another family of transcription factors regulating the fate of CD4 T cells during their differentiation are the signal transducers and activators of transcription (STATs) [63]. While STAT4 signalling is important for Th1 differentiation, STAT6 and STAT3 mainly contribute to Th2 and Th17 differentiation, respectively. Therefore, we sought to assess the phosphorylation of various STAT proteins in CD4 Tn and Tm following ISO or HC treatment and TCR stimulation. In CD4 Tn, ISO and HC reduced the phosphorylation of STAT4 and STAT6 (Figure S3A). Furthermore, Forsk only reduced pSTAT4 (Figure S3A), indicating that the effect of ISO on STAT6 is cAMP‐independent. In CD4 Tm, ISO, HC and Forsk reduced the phosphorylation of STAT4, STAT6, STAT3 and STAT5 (Figure S3A). In line with the results from LTFs, only the ratio between pSTAT4 (reflecting Th1 programme) and pSTAT6 (reflecting Th2 programme) was reduced in CD4 Tn after treatment with ISO, HC or Forsk, while no effect was observed in CD4 Tm (Figure S3B). This further consolidates the notion that the stress hormones favour the Th2 polarization, by intrinsically inhibiting the Th1 cell programme in CD4 Tn.

To confirm our observation reflected by the expression of the classic Th1, Th2 and Th17 LTFs, we further analysed the Th1, Th2 and Th17 cytokines secreted into the cell culture media, using multiplex electrochemiluminescence assays (refer to *Materials and Methods*). In line with the LTF results, the key Th1 cytokines, TNF‐α, IFN‐γ, GM‐CSF (Figure 2d) and to some extent IL‐2 (Figure S2B), were reduced in CD4 Tn, when treated with ISO or HC. On the contrary, ISO or HC reduced the secretion of Th2 cytokines, IL‐4, IL‐5 and IL‐13 in CD4 Tm, but not CD4 Tn (Figure 2d), reflecting the significant decrease in GATA3 expression in CD4 Tm, but not CD4 Tn (Figure 2a). To exclude the possibility that our cytokine observation was simply due to the observed difference in proliferation, we analysed the intracellular cytokine levels. Notably, an intracellular cytokine staining of CD4 Tn showed that ISO and/or HC significantly decreased the frequency of Th1 cytokines IFN‐γ, TNF‐α and IL‐2 (Figure 2e, f), whereas that of the Th2 cytokine IL‐4 remained unchanged and the percentages of IL‐5 only significantly decreased with HC (Figure S2C). Interestingly, we did not observe any change in the levels of Th1 cytokines among CD4 Tm (Figure 2d), although T‐bet was also significantly decreased in CD4 Tm. These results could be explained by the reported observation that the main function of T‐bet in Th1 differentiation is to inhibit GATA3 instead of positively regulating IFN‐γ expression [64]. In this scenario, Th1 cytokine expression in CD4 Tm could be maintained by other pathways even though T‐bet expression was reduced. In the culture supernatants, IL‐17 expression was slightly decreased in CD4 Tm (Figure S2B), in line with the decreased expression of RORγT and a decreased ratio of Th1/Th17 LTFs. Unexpectedly, the Th17 cytokines IL‐17 and IL‐21 remained unchanged in CD4 Tn (Figure S2B), although the Th17 master TF RORγT was decreased with ISO and HC. Interestingly, IL‐21 secretion was significantly but only slightly increased in CD4 Tm treated with HC. In CD4 Tn, IL‐10 was also slightly decreased by ISO (Figure S2B), in line with the decreased FOXP3 expression (Figure 2a).

In summary, our data show that ISO and HC have a cell‐type‐specific effect on CD4 T ‐cell differentiation, that is, inhibiting the Th1 cell programme in CD4 Tn. On the contrary, both hormones inhibit Th2 cytokine production in CD4 Tm. As no exogenous cytokines or a co‐culture with antigen‐presenting cells was employed, this unrecognized effect was regulated through a CD4 T‐cell‐intrinsic mechanism.

### Stress hormones alter mTORC1 signalling to inhibit Th1 polarization in naïve CD4 T cells

**FIGURE 3:**
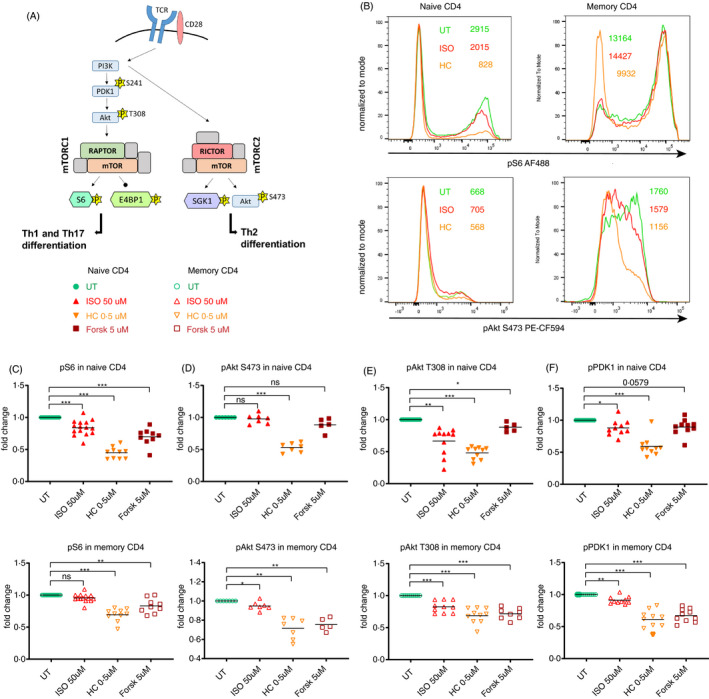
Stress hormones alter mTORC1 signalling to inhibit Th1 polarization in naïve CD4 T cells. (a) Graphical representation of a simplified view of the mTORC1 and mTORC2 involvement in the differentiation of CD4 T cells into different T‐helper subsets (adapted from Ref [87]). (b) Histograms showing the geometric mean of pS6 (S235/236) and pAkt (S473) in naïve (left) and memory (right) CD4 T cells of a representative donor. (c–f) Scatter dot plots showing the effect of ISO, HC and Forsk on the phosphorylation of different proteins of the mTOR pathways: (c) pS6 (S235/236) (*n* = 10–15), (d) pAkt (S473) (*n* = 5–7), (e) pAkt (T308) (7–11), (f) pPDPK1/pPDK1 (S241) (*n* = 10). The fold change was normalized to UT. Isoproterenol (β_2_AR agonist); hydrocortisone (HC) (synthetic glucocorticoid); UT, untreated but still stimulated; US, unstimulated. Each individual value in each group in the scatter dot plots is displayed. The horizontal bars in (c–f) represent the mean. The results in (c–f) were analysed using one‐way ANOVA with multiple comparison correction. ns, not significant; *p ≤ 0.05, **p ≤ 0.01 and ***p ≤ 0.001

The expression of the CD4 LTFs is not the only pathway regulating the CD4 T‐cell differentiation and cytokine expression. Although the whole extent of mTORC1 and mTORC2 regulation in CD4 T cells is still not fully understood, mTORC1 is considered to be crucial for Th1 and Th17 differentiation, while mTORC2 is a determinant for Th2 differentiation [65] (Figure 3a). After observing that the stress hormones differentially affected cytokine production in CD4 Tn and Tm, we hypothesized that ISO and HC might interfere with the mTOR pathway in order to differentially affect Th1 and Th2 cytokine expression. To test this hypothesis, we analysed the phosphorylation level of S6 (Ser235/236) and Akt (Ser473) as a proxy for mTORC1 and mTORC2 activity, respectively. In line with the inhibition of the Th1 programme, ISO reduced S6 phosphorylation in CD4 Tn (Figure 3b, c), while reducing Akt S473 phosphorylation in CD4 Tm (Figure 3b, d). HC reduced both pS6 S235/236 and pAkt S473 in CD4 Tn and Tm, displaying once again a more universal suppressive effect. Forsk also reduced pS6 (S235/236) in CD4 Tn and Tm, while only reducing pAkt (Ser473) in CD4 Tm, indicating the implication of cAMP signalling in the ISO‐induced inhibition of mTORC in the respective subsets. Indeed, cAMP was previously described to inhibit mTOR in mouse embryonic fibroblasts [66]. To further elucidate at which stage of the PI3K/AKT/mTOR pathway the stress hormone analogues interfere with the mTORC1/2, we examined other proteins in the pathway, namely pAkt (Thr308) and pPDK1 (Ser241). In both CD4 Tn and Tm, pAkt (Thr308) and pPDK1 (Ser241) were decreased by ISO and HC (Figure 3e, f), suggesting that both compounds, at least partially, act upstream of PI3K. Forsk also reduced pAkt (Thr308) and pPDK1 (Ser241), indicating that ISO‐induced cAMP might interfere with the mTORC signalling pathway upstream of PI3K (Figure 3e, f). In summary, we showed that ISO inhibits mTORC1 selectively in CD4 Tn, while inhibiting mTORC2 in CD4 Tm. This effect is at least partially mediated via cAMP signalling acting upstream of PI3K, since Forsk, a cAMP inducer, has a similar impact on mTOR signalling. On the contrary, HC has a more generally suppressive mode of action, inhibiting both mTORC1 and mTORC2 upstream of PI3K.

### Stress hormones induce the expression of *PER1* to inhibit Th1 cytokine expression via mTORC1 in naïve CD4 T cells

**FIGURE 4:**
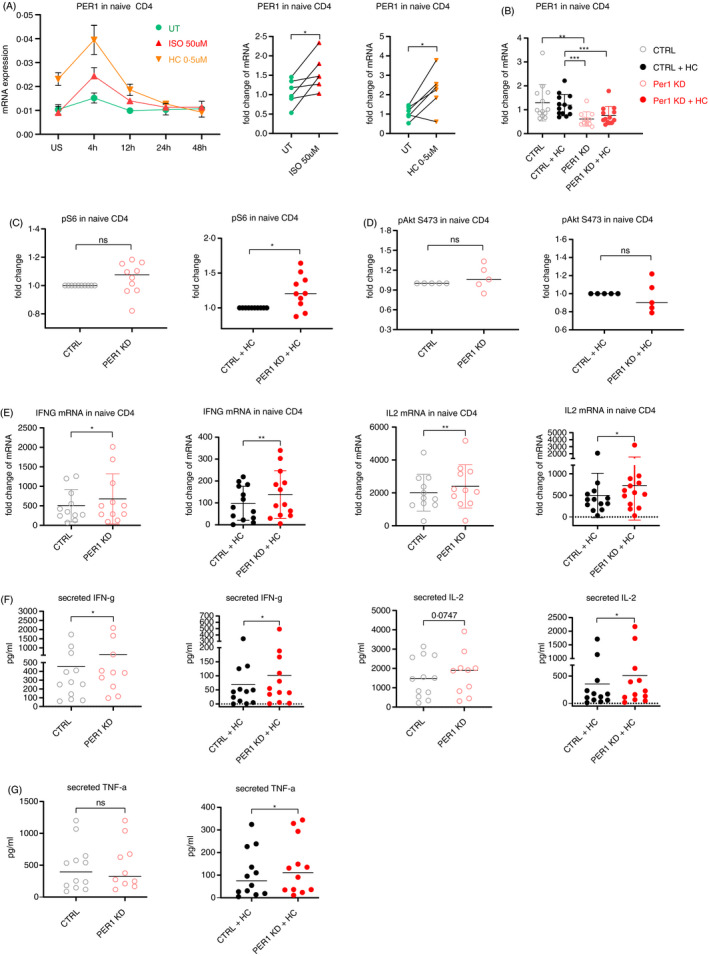
Stress hormones induce the expression of *PER1* to inhibit Th1 cytokine expression via mTORC1 in naïve CD4 T cells. (a) mRNA expression of the clock genes *PER1* in naïve CD4 following ISO or HC treatment and TCR stimulation. Far left: time course over the first 48h of a representative donor. Middle and far right: Graphs showing the pooled results of several donors for the mRNA expression of *PER1* after 4 h of ISO (red) or HC (orange) treatment. (b), The mRNA expression of PER1 following *PER1*‐specific siRNA or scrambled control (CTRL) (grey/black) siRNA knockdown in the presence (full circles) or absence (empty circles) of HC in naïve CD4 (*n* = 11–13). (c, d) The fold change of S6 phosphorylation (S235/236) (c, *n* = 10) and pAkt (S473) (d, *n* = 5) in naïve CD4 T cells following *PER1*‐specific or CTRL siRNA knockdown and TCR stimulation in the presence or absence of HC. (e) The mRNA expression of IFNG and IL‐2 following *PER1*‐specific siRNA or scrambled control (CTRL) (grey/black) siRNA knockdown in the presence (full circles) or absence (empty circles) of HC in naïve CD4 (*n* = 11–13). (f, g) Concentration of secreted IFN‐γ and IL‐2 (f) as well as TNF‐α (g) in the cell culture medium of naïve CD4 T cells following ISO or HC treatment and 48‐h TCR stimulation (*n* = 10–12). Isoproterenol (β_2_AR agonist); hydrocortisone (HC) (synthetic glucocorticoid); UT, untreated but still stimulated; US, unstimulated. Each individual value in each group in the scatter dot plots was displayed. The results in (a–g) were analysed using the paired *t*‐test. ns, not significant; *p ≤ 0.05, **p ≤ 0.01 and ***p ≤ 0.001. The horizontal bars in (c, d, f, g) represent the mean. In b, e, data are represented as mean ± standard deviation (SD). See also Figures S4–S6

mTOR exhibits circadian oscillations and has a reciprocal regulatory relationship with the circadian proteins in other cell types [67, 68, 69, 70]. Furthermore, it is well documented that there is a strong interaction between stress hormones and the circadian rhythm machinery, especially in one of the critical immune functions [14], for example trafficking of both monocytes and lymphocytes [71, 72]. Specific circadian rhythm genes have also been shown to regulate Th2 [18] or Th17 differentiation [17]. Therefore, we sought to study whether and how the molecular circadian machinery might be involved in the stress hormone‐mediated mTORC1‐dependent CD4 T‐cell differentiation. To identify which specific components of the circadian machinery could be involved in this process, we first measured the dynamic expression pattern of various mammalian circadian rhythm genes, following ISO or HC treatment and TCR stimulation. Only three out of nine genes were consistently altered at 4 h following ISO or HC treatment in CD4 Tn, namely *PER1*, *PER2* and *PER3* (Period Circadian Regulator 1, 2, 3) (Figures 4a, S4A, B and S5A, B). While *PER1* was significantly increased (Figure 4a), the expression of *PER2* and *PER3* was modestly or significantly reduced (Figure S5A, B). No consistent pattern for any of the analysed circadian genes was observed in CD4 Tm (Figure S4B). Indeed, others have also observed that these PER genes are induced by noradrenaline and/or glucocorticoid in either total CD4 T cells, peripheral blood mononuclear cells or the liver [73, 74, 75]. Furthermore, glucocorticoids have been shown to induce *PER1* expression by directly binding to glucocorticoid receptor‐binding sites near the transcription start site (TSS) of *PER1* in a human epithelial cell line [76]. Therefore, we first investigated the potential involvement of *PER1* in the mTORC1‐dependent CD4 T‐cell differentiation.

To examine whether the upregulation of *PER1* is able to inhibit the mTOR pathway, we knocked down *PER1* in CD4 Tn and Tm before treating them with HC and assessed the mTORC1 and mTORC2 activity, by staining pS6 (S235/236) and pAkt (S473), respectively. HC was selected here over ISO, because HC was able to induce higher levels of *PER1* expression, compared with ISO (Figure 4a). Confirming our hypothesis, *PER1* knockdown (KD) in CD4 Tn (Figure 4b) partially rescued the HC‐induced inhibition of mTORC1, shown by a significant increase in pS6 after *PER1* KD and under HC treatment (8/10 donors) (Figure 4c). Further reducing the already low basal levels of *PER1* with siRNA without HC treatment already increased pS6 in 5 of 10 tested donors (Figure 4c). On the contrary, *PER1* KD did not alter the activity of mTORC2 (pAkt S473) in CD4 Tn (Figure 4d) and had no impact on either mTORC1 or mTORC2 in CD4 Tm (Figure S6A, B). As mTORC1 critically regulates Th1 differentiation [65], our results show that *PER1* is an inhibitor of mTORC1 in CD4 Tn, indicating that *PER1* thus regulates Th1 differentiation.

To examine whether the inhibition of mTORC1 by *PER1* indeed contributes to the repression of the Th1 programme, we knocked down *PER1* in CD4 Tn and Tm before treating them with HC and measured the expression and secretion of Th1 cytokines. Abolishing the upregulation of *PER1* using specific *PER1* siRNA (*PER1* KD) (Figure 4b; Figure S6A) partially rescued the HC‐induced inhibition of the Th1 cytokine genes, *IFNG* and *IL2* in CD4 Tn (Figure 4e), while having no impact on CD4 Tm (Figure S6C). Encouragingly, *PER1* KD without the presence of HC also increased the mRNA expression of *IFNG* and *IL2* by further reducing the already low expression of *PER1* at baseline (Figure 4e). In agreement with the mRNA data, *PER1* KD increased the protein secretion of Th1 cytokines, such as IFN‐γ and IL‐2 (Figure 4f) in the presence of HC. In the absence of HC, IFN‐γ secretion was also significantly enhanced and IL‐2 secretion also showed a trend to be increased (*p* = 0.075) by *PER1* KD. Furthermore, *PER1* KD significantly enhanced the secretion of TNF‐α, in the presence, but not in the absence, of HC (Figure 4g).

As *PER1* KD only partially rescued the HC‐induced effects on Th1 differentiation, we asked whether *PER2* and *PER3*, the only consistently downregulated clock genes following ISO or HC treatment, might also contribute to the regulation of CD4 T‐cell differentiation (Figure S5A, B). To this end, we knocked down *PER2* and *PER3* in CD4 Tn using specific siRNA, either alone or in combination, and assessed the mRNA expression of the Th1 cytokines, IFNG and IL‐2. The different siRNAs specifically decreased the expression of the targeted PER gene without affecting the expression of the other PER genes (Figure S5C). However, neither *PER2* KD, nor *PER3* KD, nor the combination of both was sufficient to reduce the expression of Th1 genes (Figure S5D). This indicates that *PER2* and *PER3* only endure a bystander effect of ISO and HC treatment, but are not involved in the inhibition of Th1 differentiation.

In summary, our data lay out a novel CD4 Tn‐intrinsic mechanism, through which stress hormones modulate the T‐helper cell polarization in CD4 Tn via inducing *PER1* expression and inhibiting the mTORC1 pathway (Figure S7).

## DISCUSSION

Here, we showed that both β_2_AR‐ and GR‐mediated stress signalling inhibit Th1 polarization and cytokine expression in CD4 Tn via the interplay between the circadian gene *PER1* and the mTORC1 signalling pathway. In line with our observation, previous studies have shown that *PER1* is able to inhibit Akt/mTOR signalling, although in squamous cell carcinoma, [70] and plays a role in the regulation of cytokine expression, such as IFNG, in NK cells [77]. Here, we linked those separate observations within primary naïve CD4 T cells. We demonstrated that upon stress hormone signalling, *PER1* is induced and able to inhibit mTORC1 signalling to regulate the expression of Th1 cytokines. In short, we revealed a novel naïve CD4 T‐cell‐intrinsic mechanism through which the stress hormones impede Th1 differentiation, consequently shifting the T‐helper subset balance towards Th2. Although a very similar effect was observed for both ISO/β_2_AR signalling and HC/GR signalling on suppressing Th1 polarization, a stronger effect was noticed via GR signalling in different aspects. Furthermore, GR signalling seemed to suppress Th2 response in both naïve and memory CD4 T cells while β_2_AR signalling only suppressed Th2 response in memory CD4 T cells. Taken together, our data demonstrated that β_2_AR agonists inhibit Th1/Th2 programme balance only in naïve CD4 T cells, whereas activating GR signalling suppresses that ratio in both naïve and memory CD4 T cells.

Our work adds a new layer of understanding to the current paradigm that shifting the Th1/Th2 balance is dependent on adrenergic receptor signalling in DCs to reduce the DC‐derived IL‐12 expression [37, 39]. As a robust Th2 response is required for a high production of antibodies, the increased Th2 response during stress responses should lead to higher levels of secreted antibodies. Indeed, B cells exposed to a stress hormone increased the production of different classes of immunoglobulins, depending on the experimental or disease context [78 ]. Another example showing that the effect of stress hormones on T cells converges with that of other cells to mediate a type II immune response was described previously [41, 79]. As demonstrated by Tracey and colleagues, noradrenaline released by the splenic nerves induces choline acetyltransferase (ChAT)‐expressing T cells to synthesize and release acetylcholine, which in turn inhibits the expression of inflammatory cytokines in macrophages [79] and promotes the production of plasma cells [41]. These studies, together with our work, showcase that during the immunological chain of events from antigen uptake/presentation to T‐cell differentiation, and further to antibody secretion in B cells, the existence of each cell‐type‐specific/‐intrinsic effect induced by stress hormones converges with the others to specifically boost one arm (i.e. Th2) of the immune system. These overlapping mechanisms make it clear that the stress hormones act in parallel on different immune cells to meticulously regulate the context‐specific immune responses.

Our cell‐type‐specific findings in CD4 Tn significantly complement the current understanding of how stress hormones are able to favour Th2 responses via suppressing Th1 differentiation in CD4 Tn, rather than only causing a universal immunosuppressive effect. On the contrary, we showed that the stress hormones have an opposite effect in CD4 Tm, reducing the production of Th2 cytokines IL‐4, IL‐5 and IL‐13 via an mTORC2‐dependent mechanism. The related discrepancy between distinct immune subpopulations has already been observed previously, describing that naïve and different effector CD4 T subsets respond in different ways to β_2_AR signalling [25, 80]. This effect was attributed to differential β_2_AR expression levels and/or the stage of cell differentiation. However, those studies did not investigate the CD4 T‐cell‐intrinsic mechanisms through which stress hormones regulate CD4 T‐cell differentiation. More recently, noradrenaline has been shown to preferentially modulate the function of memory CD8 T cells, due to a higher sensitivity to noradrenaline, based on a higher expression of β_2_AR [81]. As a G protein‐coupled receptor (GPCR), the β_2_AR signals through G proteins. However, depending on which specific G protein subunit is coupled to the receptor, a different downstream pathway might be induced. It has shown that human T cells alter their G protein subunit repertoire during differentiation [82], which could at least partially account for the differential effect of noradrenaline/norepinephrine analogues on naïve/memory T‐cell subpopulations, as we demonstrated here.

As the stress hormones are a driving force not only in stress responses but also in the regulation of the circadian rhythm, we and others hypothesized that the circadian signalling might play an important role in regulating the immune system. Noradrenaline/norepinephrine and glucocorticoids have been shown to control lymphocyte trafficking during the 24h cycle via the molecular circadian clock machinery [72, 83, 84]. Moreover, emerging evidence attributes specific functions to specific clock genes in the context of distinct immune responses in different cell types (reviewed here [14, 15, 16]). Together with a recent report showing that the central clock gene BMAL1 is dispensable for T‐cell functions [85], our study suggests that it is the specific circadian genes, instead of the core/master circadian regulators (BMAL1 and CLOCK), that regulate specific CD4 Tn responses. For the first time, we were able to show that the circadian rhythm gene *PER1* inhibits the expression of Th1 cytokines by reducing mTORC1 signalling in CD4 Tn. At the same time, stress hormone signalling also directly inhibits mTOR signalling by affecting the PI3K/AKT pathway. These two parallel mechanisms are probably accompanied by other regulatory pathways, such as NFAT, NF‐κB and MAPK, already known to be affected by β_2_AR and GC signalling via genomic or non‐genomic mechanisms [27, 28, 29, 30]. We would like to point out that although the flow cytometry antibodies we used in this work were all purchased from leading suppliers and were often independently validated by many other reports, we might still benefit from additional independent validation of non‐flow cytometry approaches.

The circadian machinery is dysregulated in many complex diseases, including various immune‐associated diseases [21, 22]. Targeting the circadian machinery could be a potent, although challenging, new avenue to treat some of those diseases. It is worthy to note that the circadian clock also plays a role in an optimal vaccination response [86]. Due to the tight and dynamic regulation of the molecular clock machinery, more research has to be performed to further characterize the role of the different circadian rhythm genes in specific immune responses of various immune subsets. Our study contributes to this cause by identifying novel pathways through which stress hormones intrinsically inhibit Th1 differentiation in naïve CD4 T cells via the specific circadian clock gene *PER1* and the mTORC1 signalling pathway. Last but not least, as our work is fully based on primary human T cells, the translational potential is evident.

## CONFLICT OF INTEREST

The authors declare no competing interests.

## AUTHOR CONTRIBUTIONS

C.M.C. designed and performed experiments. C.M.C. analysed the data and wrote the manuscript. A.C. contributed to the project in the context of her master thesis. A.B., N.Z., K.G. and T.A. performed some experiments and gave technical advice. A.S., M.O. and F.Q.H. supervised the project. F.Q.H. oversaw the project and revised the manuscript. All the authors read and edited the manuscript.

## ETHICAL APPROVAL

Buffy coats from healthy donors were obtained via the Red Cross Luxembourg, under strict ethical regulation, data protection. The study protocol has been approved by the ethical committee of Red Cross Luxembourg. Informed consent was obtained from each donor before blood sampling.

## Supporting information

Fig S1‐S7

Table S1‐S3
