# Supplementary material for: Stress hormone signalling inhibits Th1 polarization in a CD4 T‐cell‐intrinsic manner via mTORC1 and the circadian gene PER1
Source: Immunology. 2022 Mar 2;165(4):428–44. doi: 10.1111/imm.13448 (PMC9426625; doi:10.1111/imm.13448)
Supplement: Supplementary file 1 — Fig S1‐S7 [file IMM-165-428-s001.docx]

# Stress hormone signaling inhibits Th1 polarization in a CD4 T-cell-intrinsic manner via mTORC1 and the circadian gene *PER1*

Christophe M. Capelle^1,2^, Anna Chen^1^, Ni Zeng^1,2^, Alexandre Baron^1^, Kamil Grzyb^3^, Thais Arns^3^, Alexander Skupin^3^, Markus Ollert^1,4^, Feng Q. Hefeng^1,5,*^

^1^ Department of Infection and Immunity, Luxembourg Institute of Health (LIH), 29, rue Henri Koch, L-4354, Esch-sur-Alzette, Luxembourg

^2^ Faculty of Science, Technology and Medicine, University of Luxembourg, 2, avenue de Université, L-4365, Esch-sur-Alzette, Luxembourg

^3^ Luxembourg Centre for Systems Biomedicine (LCSB), University of Luxembourg, 6, avenue du Swing, L-4367, Belvaux, Luxembourg

^4^ Department of Dermatology and Allergy Center, Odense Research Center for Anaphylaxis (ORCA), University of Southern Denmark, Odense, 5000 C, Denmark

^5^ Institute of Medical Microbiology, University Hospital Essen, University of Duisburg-Essen, D-45122, Essen, Germany

* Corresponding author. Direct correspondence to [feng.he@lih.lu](mailto:feng.he@lih.lu)

## Seven Supplementary Figures and Legends


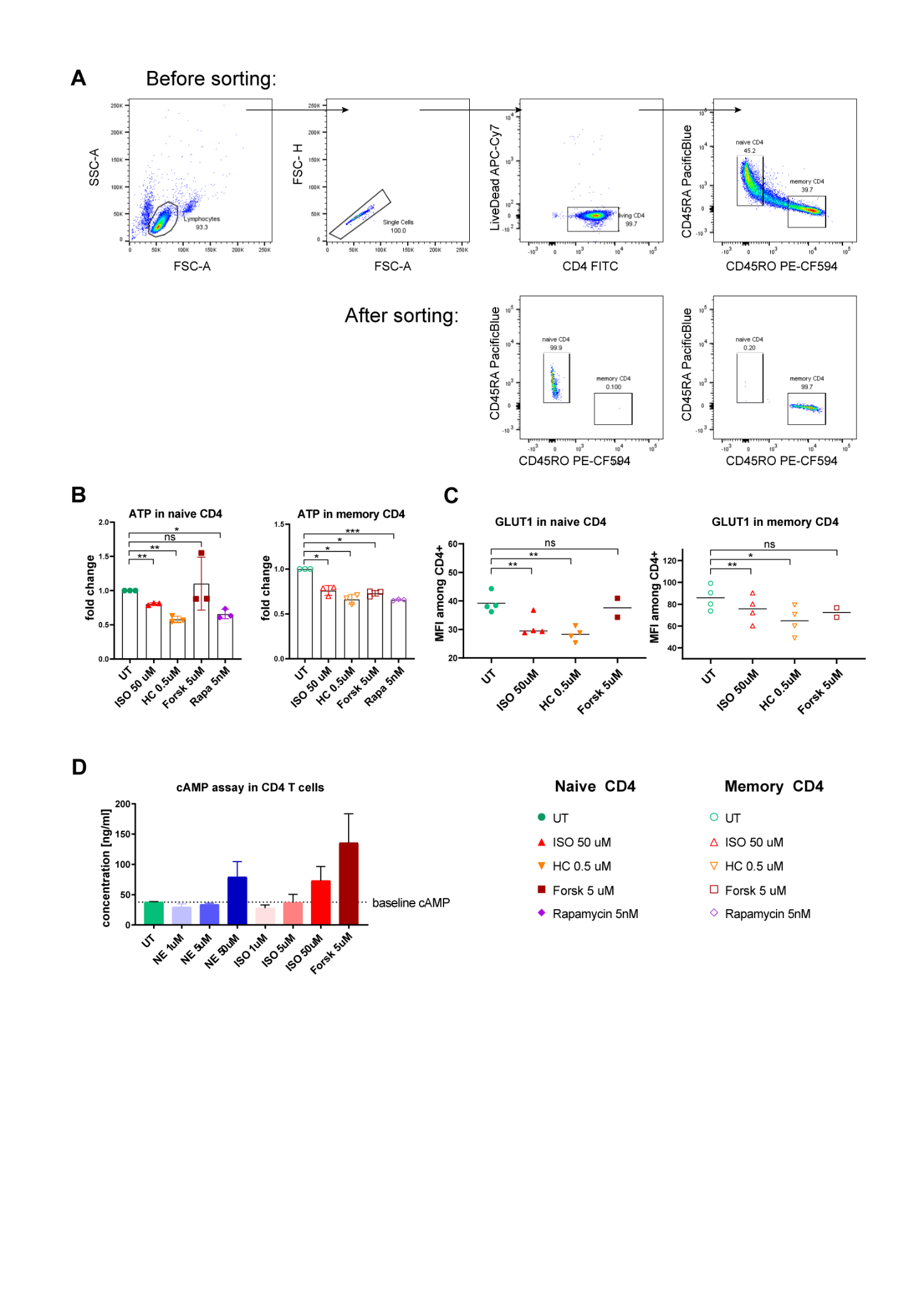


### Figure S1: Sorting efficiency and additional measured markers for the metabolic activity phenotype.

(**A**) Flow cytometry plots showing the sorting efficiency of naïve and memory CD4 T cells with a purity higher than 99%. The plot in the left and right represent naïve or memory CD4 T cells in the row “After sorting”, respectively. Of note, the CD25 gate before the CD45RA versus CD45RO gate was not displayed here due to limited space. (**B**) Fold change in intracellular ATP concentration following treatments with ISO, HC, Forsk and Rapamycin and 48h TCR stimulation in naïve (left) and memory (right) CD4 T cells (n=3). The values were normalized to the UT samples. (**C**) Geometric mean (MFI) of GLUT1 in naïve (left) and memory (right) CD4 following treatment with ISO, HC or Forsk and 48h TCR stimulation (n=2-4). (**D**) cAMP assay showing the induction of intracellular cAMP levels in total CD4 T cells following treatments with Norepinephrine (NE), ISO or Forsk in different concentrations. Isoproterenol (β_2_AR agonist); hydrocortisone (HC) (synthetic glucocorticoid); UT, untreated but still stimulated. The results in (B-D) were analyzed using one-way ANOVA with multiple comparison correction. ns or unlabeled, not, significant; *p<=0.05, **p<=0.01, and ***p<=0.001. The horizontal bars or boxes in (C) represent the mean. Data in B and D are represented as mean ± standard deviation (s.d.).


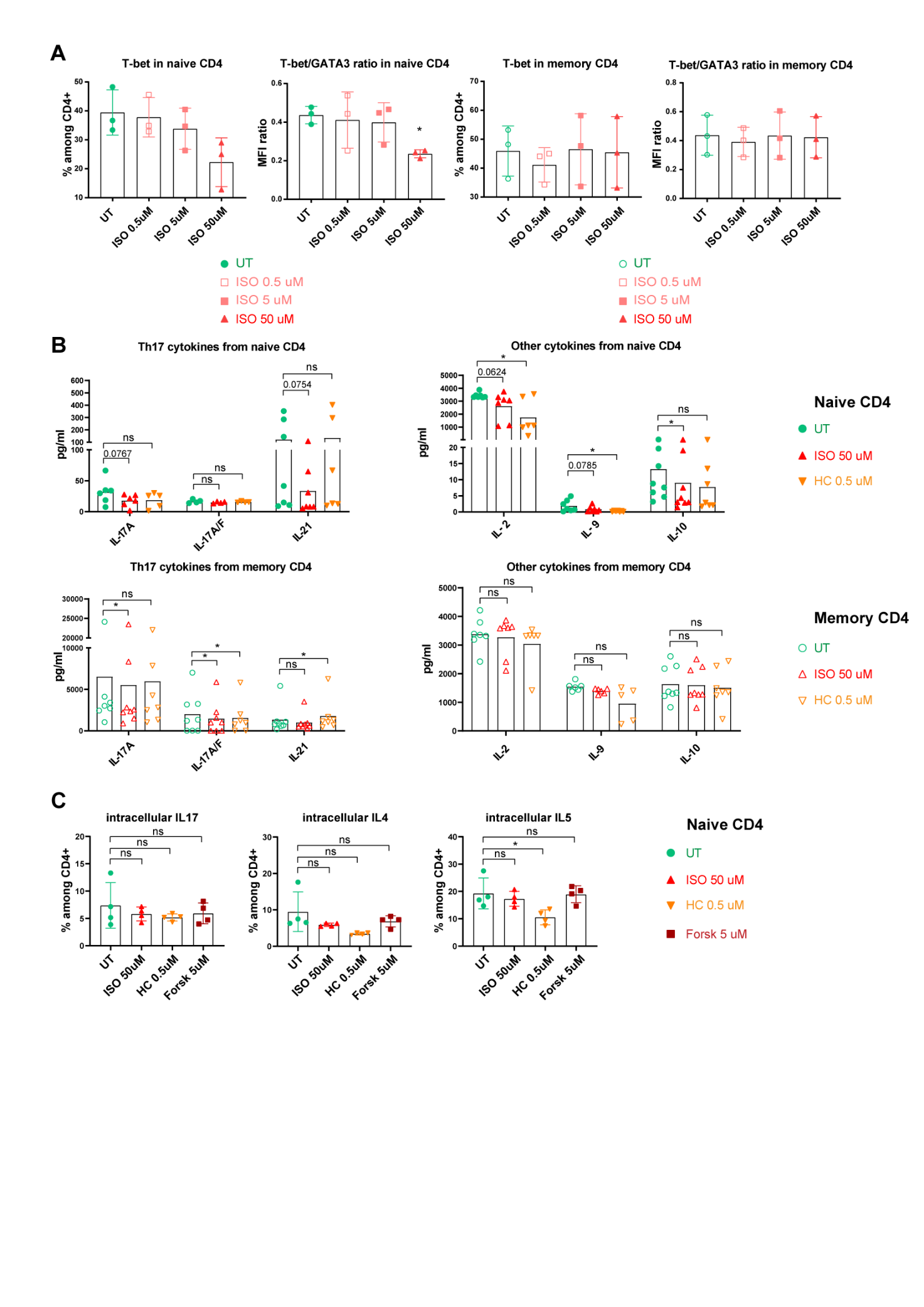


### Figure S2: Extended analysis on the secretion of Th17 and other cytokine and production of intracellular cytokines.

(**A**) Expression of T-bet and the T-bet/GATA3 ratio in naïve or memory CD4 T cells treated with different concentrations of ISO followed by 48h TCR stimulation (n=3). (**B**) Secreted cytokines, measured in the culture medium after 48h of TCR stimulation with the MSD multiplex assays. Cytokines secreted by naïve CD4 T cells are shown in the top row, and memory CD4 in the bottom row. Th17 cytokines (left) and others (right) are grouped in different graphs (n=6-8). (**C**) Graphs showing the levels of various intracellular cytokines in naïve CD4 after ISO, HC or Forsk treatment and 48h TCR stimulation (n=4). Isoproterenol (β_2_AR agonist); hydrocortisone (HC) (synthetic glucocorticoid); UT, untreated but still stimulated. The results in (A-C) were analyzed using one-way ANOVA with multiple comparison correction. ns or unlabeled, not significant; *p<=0.05, **p<=0.01, and ***p<=0.001. The boxes in (B) represent the mean. Data in A and C are represented as mean ± standard deviation (s.d.).


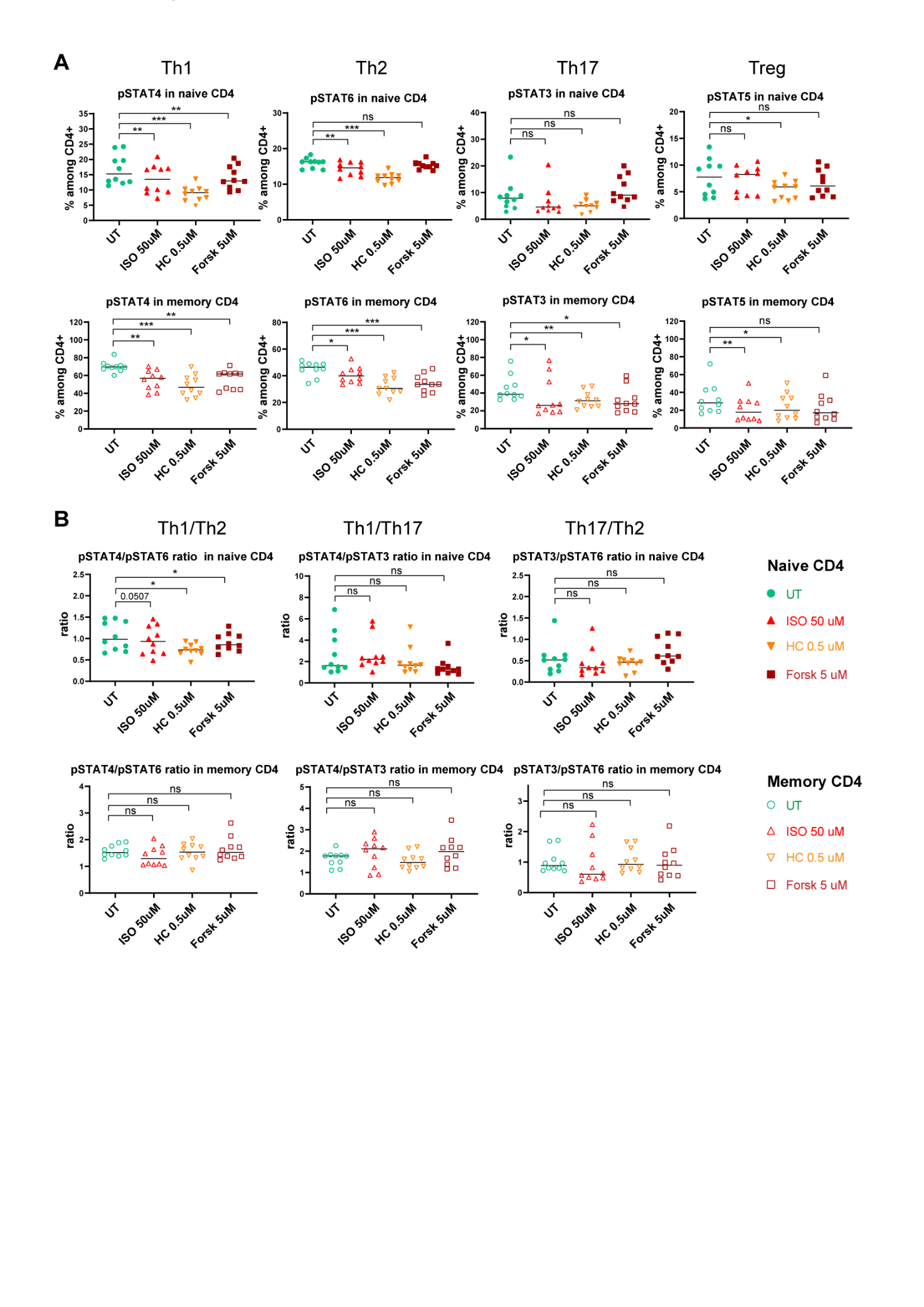


### Figure S3: STAT profile is also shifted to reduce Th1 polarization in naive CD4.

(**A**) Graphs showing the expression of various phosphorylated STAT proteins in naïve (top) and memory (bottom) CD4 T cells following ISO, HC or Forsk treatment and 48 h TCR stimulation (n=10). (**B**) Graphs showing the ratio between the percentages of cells expressing different phosphorylated STATs among living CD4 T cells to assess the Th1/Th2/Th17 balance in naïve (top) and memory (bottom) CD4 (n=10). Isoproterenol (β_2_AR agonist); hydrocortisone (HC) (synthetic glucocorticoid); UT, untreated but still stimulated. The results in (A-B) were analyzed using one-way ANOVA with multiple comparison correction. ns or unlabeled, not significant; *p<=0.05, **p<=0.01, and ***p<=0.001. Each individual value in each group in the scatter dot plots was displayed. The horizontal lines in (A, B) represent the mean.


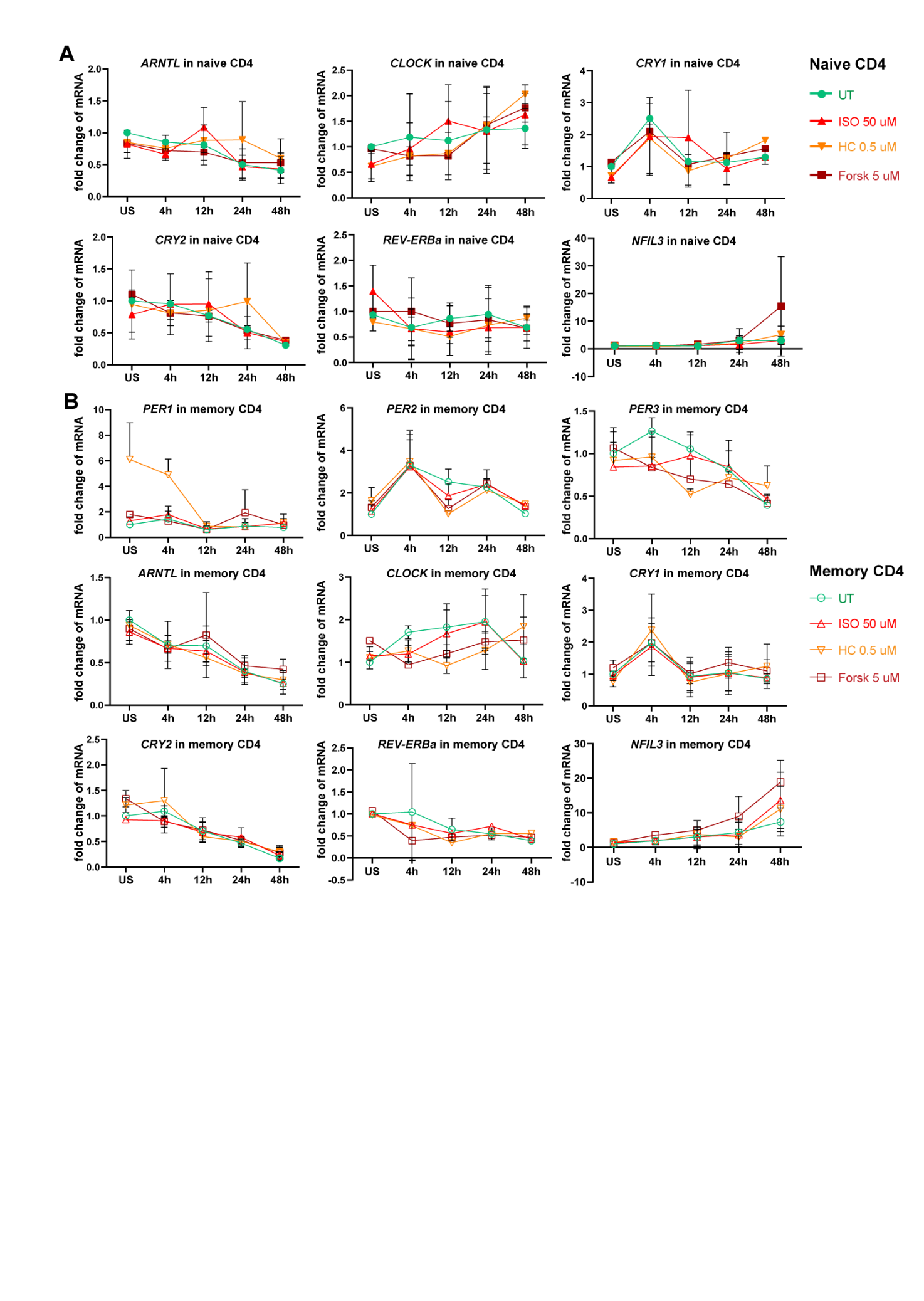


### Figure S4: Circadian rhythm gene expression in naive and memory CD4 T cells following TCR stimulation and stress hormone treatments.

(**A**), (**B**) 48h-timecourse of mRNA expression of circadian clock genes in naïve (**A**) or memory (**B**) CD4 following ISO or HC treatment and TCR stimulation (n=3-6). Isoproterenol (β2AR agonist); hydrocortisone (HC) (synthetic glucocorticoid); UT, untreated but still stimulated; US, unstimulated. For each time point, the mean and standard deviation is represented. The data at each time point in each plot was presented as mean ± standard deviation (s.d.). Line was used to connected different time points.


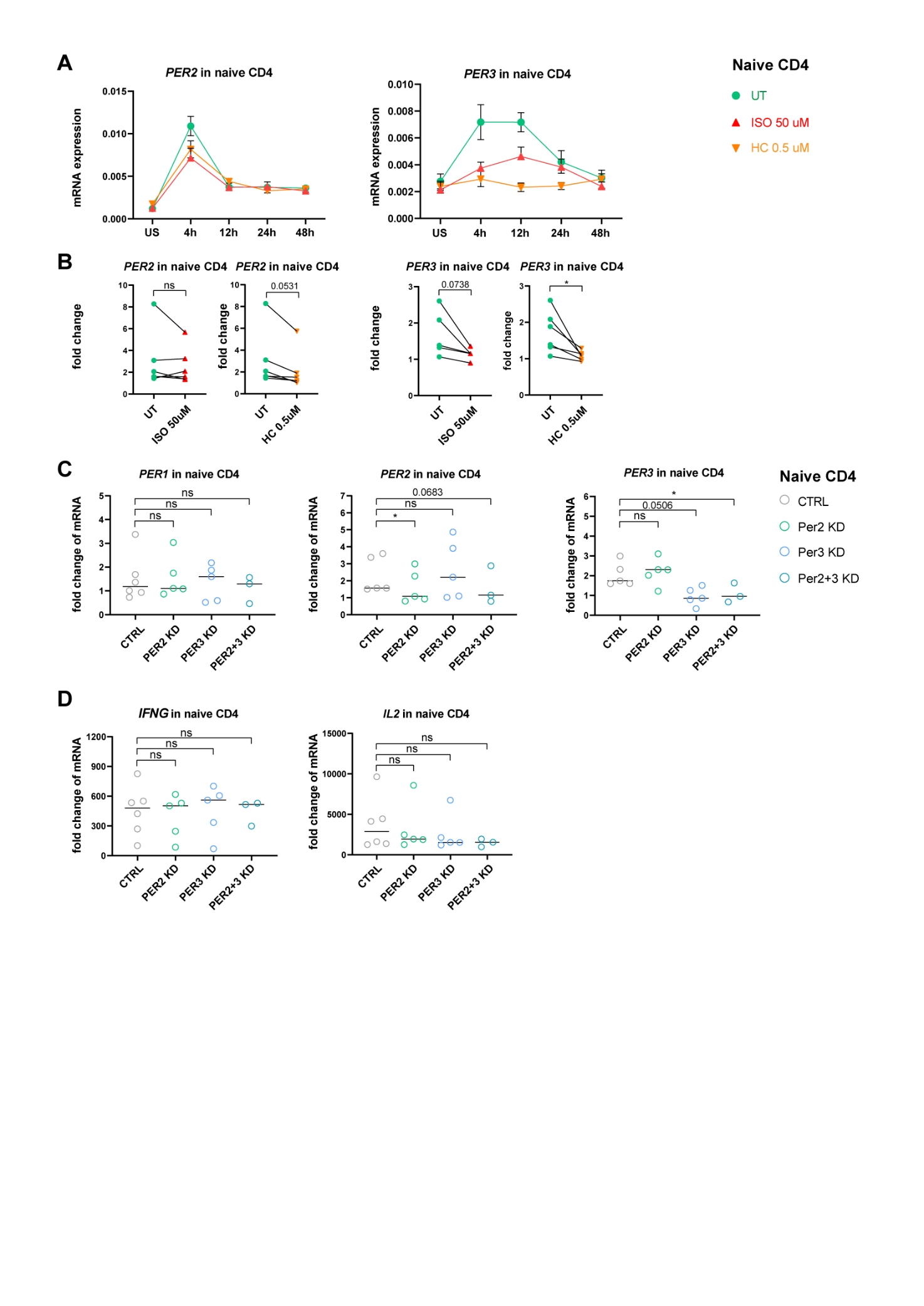


### Figure S5: *PER2* and *PER3* have no impact on Th1 gene expression in naive CD4 T cells.

**(A, B)** mRNA expression of the clock genes *PER2 and PER3* in naïve CD4 following ISO or HC treatment and TCR stimulation. Time course over the first 48h of a representative donor (**A**). The data at each time point in each plot was presented as mean ± standard deviation (s.d.). Line was used to connected different time points. Graphs showing the pooled results of several donors for the mRNA expression of *PER2 and PER3* after ISO (red) or HC (orange) treatment (**B**). (**C**) mRNA expression of different period circadian genes in naïve CD4 T cells following *PER2* and/or *PER3* siRNA knockdown and 24h TCR stimulation (n=3-6). (**D**) mRNA expression of Th1 genes in naïve CD4 T cells following *PER2* and/or *PER3* siRNA knockdown and 24h TCR stimulation (n=3-6). CTRL, non-specific control siRNA; UT, untreated but still stimulated. The results in (A-B) were analyzed using two-way ANOVA with multiple comparison correction. (C-D) were analyzed using one-way ANOVA with multiple comparison correction. ns or unlabeled, not significant; *p<=0.05, **p<=0.01, and ***p<=0.001. Each individual value in each group in the scatter dot plots was displayed. The horizontal bars (C-D) represent the mean.


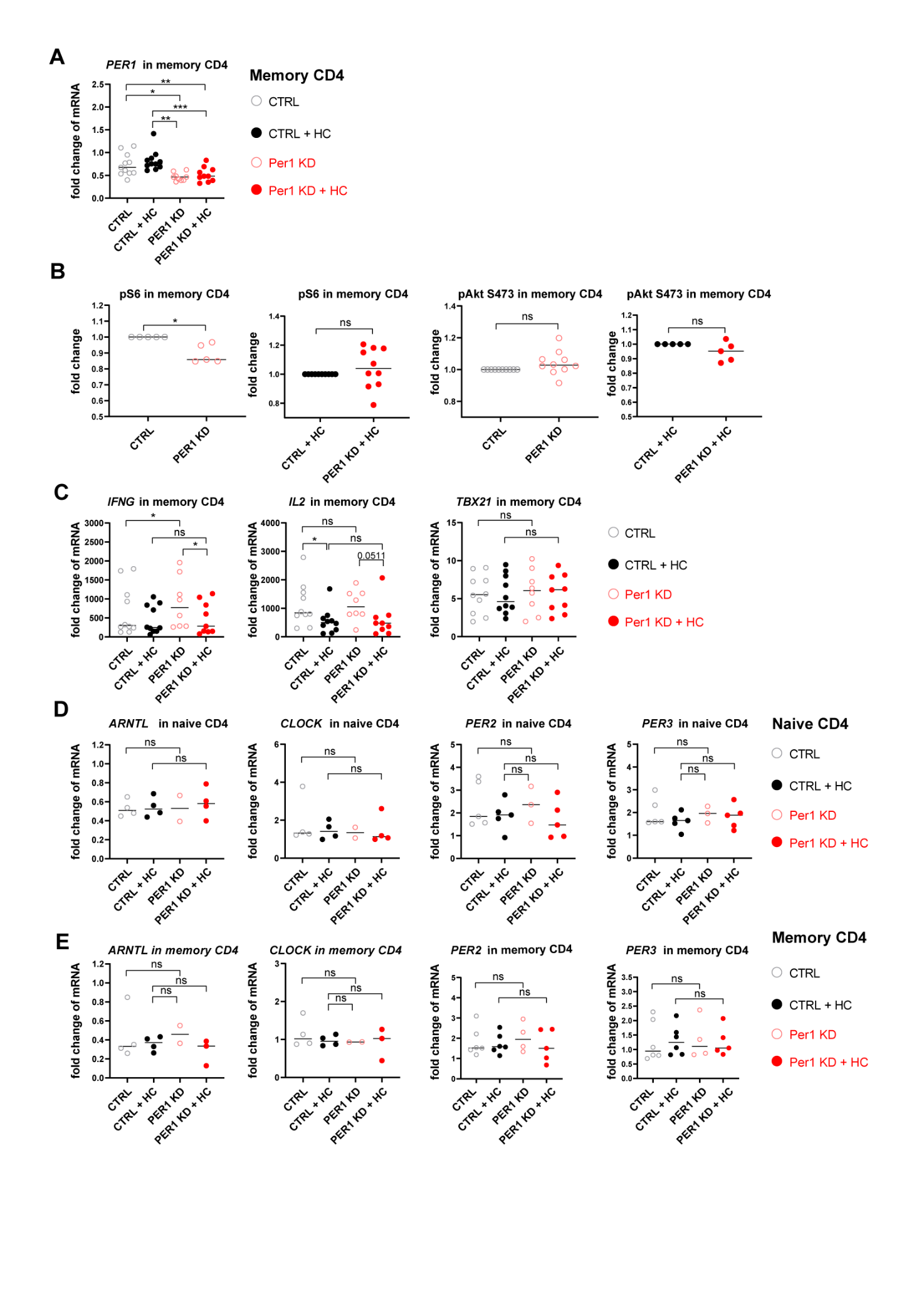


### Figure S6: *PER1* knockdown has no impact in memory CD4 T cells and on the expression of other circadian rhythm genes in naïve CD4 T cells.

(**A**), (**C**) mRNA expression of PER1 (**A**) and Th1 genes (**C**) in memory CD4 following *PER1*-specific or non-specific scrambled control (CTRL) siRNA knockdown in the presence or absence of HC (n=8-9). (**B**) The fold change in the geometric mean (also known as Geomean) of pS6 (S235/236) (n=10) and pAkt (S473) (n=5) in memory CD4 T cells following *PER1* or CTRL siRNA knockdown and TCR stimulation in the presence or absence of HC. Hydrocortisone (HC) (synthetic glucocorticoid). The fold change was normalized to CTRL. (**D**), (**E**) mRNA expression of different circadian rhythm genes in naïve (**D**) or memory (**E**) CD4 following *PER1*-specific or CTRL siRNA knockdown in the presence or absence of HC (n=2-4). The results in (A-E) were analyzed using paired t test. ns or unlabeled, not significant; *p<=0.05, **p<=0.01, and ***p<=0.001. Each individual value in each group in the scatter dot plots was displayed. The horizontal bars (A-E) represent the mean.


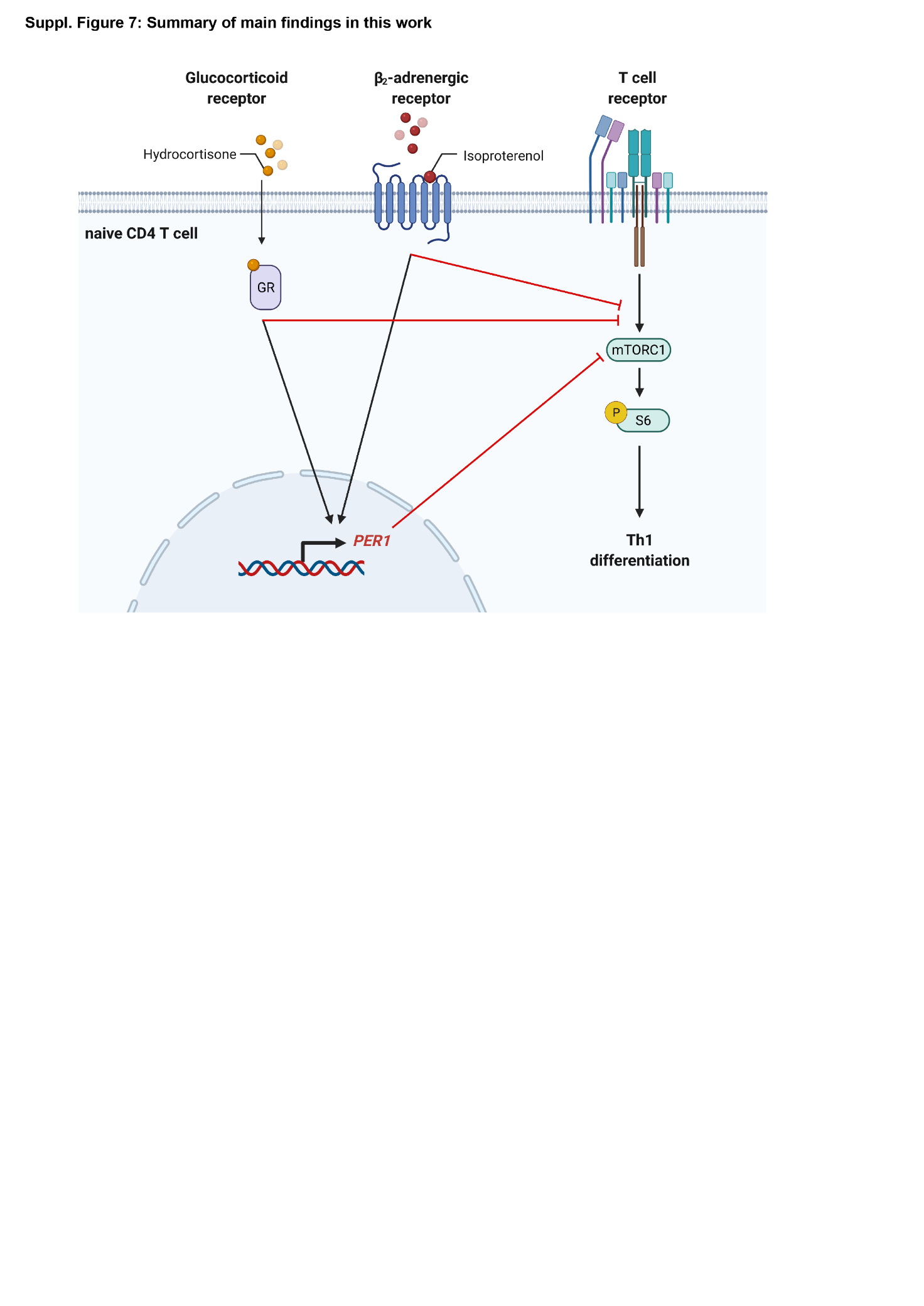


### Figure S7: Graphical summary of our main findings.

Graphical representation of findings shown in this manuscript. Stress hormone signaling inhibits T-bet and IFN-γ expression in naïve CD4 T cells via inducing the expression of *PER1* and inhibiting mTORC1 signaling, thus inhibiting Th1 differentiation. Red lines indicate inhibitory effects, whereas the black lines represent inducing effects. Isoproterenol (ISO, β_2_AR agonist); glucocorticoid receptor (GR); hydrocortisone (HC) (synthetic glucocorticoid); TCR, T cell receptor; period circadian regulator 1 (*PER1*).
