# Supplementary material for: Stress hormone signalling inhibits Th1 polarization in a CD4 T‐cell‐intrinsic manner via mTORC1 and the circadian gene PER1
Source: Immunology. 2022 Mar 2;165(4):428–44. doi: 10.1111/imm.13448 (PMC9426625; doi:10.1111/imm.13448)
Supplement: Supplementary file 2 — Table S1‐S3 [file IMM-165-428-s002.docx]

# Stress hormone signaling inhibits Th1 polarization in a CD4 T-cell-intrinsic manner via mTORC1 and the circadian gene *PER1*

Christophe M. Capelle^1,2^, Anna Chen^1^, Ni Zeng^1,2^, Alexandre Baron^1^, Kamil Grzyb^3^, Thais Arns^3^, Alexander Skupin^3^, Markus Ollert^1,4^, Feng Q. Hefeng^1,5,*^

^1^ Department of Infection and Immunity, Luxembourg Institute of Health (LIH), 29, rue Henri Koch, L-4354, Esch-sur-Alzette, Luxembourg

^2^ Faculty of Science, Technology and Medicine, University of Luxembourg, 2, avenue de Université, L-4365, Esch-sur-Alzette, Luxembourg

^3^ Luxembourg Centre for Systems Biomedicine (LCSB), University of Luxembourg, 6, avenue du Swing, L-4367, Belvaux, Luxembourg

^4^ Department of Dermatology and Allergy Center, Odense Research Center for Anaphylaxis (ORCA), University of Southern Denmark, Odense, 5000 C, Denmark

^5^ Institute of Medical Microbiology, University Hospital Essen, University of Duisburg-Essen, D-45122, Essen, Germany

* Corresponding author. Direct correspondence to [feng.he@lih.lu](mailto:feng.he@lih.lu)

## Supplemental Tables

### Supplemental Table S1. Antibodies used for human CD4 T-cell sorting.

| **Target** | **Fluorochromes** | **Dilution** | **Company** | **Clone** | **Reference** |
| --- | --- | --- | --- | --- | --- |
| CD4 | FITC | 1:20 | BD Biosciences | RPA-T4 | 555346 |
| CD25 | APC | 1:20 | BD Biosciences | M-A251 | 555434 |
| CD45RA | Pacific Blue | 1:20 | BioLegend | HI100 | 304118 |
| CD45RO | PE-CF594 | 1:20 | BD Biosciences | UCHL1 | 562299 |
| Live/Dead | Near Infra-Red | 1:500 | Thermo Fisher Scientific | N.A. | L10119 |

### Supplemental Table S2. List of antibodies used in the current study.

| **Target** | **Fluorochromes** | **Dilution** | **Company** | **Clone** | **Reference** |
| --- | --- | --- | --- | --- | --- |
| **Surface markers** | | | | | |
| CD4 | BUV395 | 1:100 | BD Biosciences | RPA-T4 | 564724 |
| CD278 (ICOS) | BV605 | 1:50 | BioLegend | C398.4A | 313538 |
| CD279 (PD-1) | BV605 | 1:50 | BioLegend | EH12.2H7 | 329924 |
| GLUT1 | AF647 | 1:50 | BD Biosciences | 202915 | 566580 |
|  |  |  |  |  |  |
| Live/Dead | Near Infra-Red | 1:500 | Thermo Fisher Scientific | N.A. | L10119 |
| **Intracellular markers** | | | | | |
| Ki-67 | AF488 | 1:50 | BD Biosciences | B56 | 561165 |
| cMyc | AF488 | 1:50 | Cell Signaling Technology | D84C12 | 12855S |
| HIF-1α | AF647 | 1:50 | BD Biosciences | 54/HIF-1α | 565924 |
| T-bet | PE | 1:50 | BioLegend | 4B10 | 644810 |
| RORγT | BV650 | 1:20 | BD Biosciences | Q21-559 | 563424 |
| FOXP3 | AF647 | 1:50 | BioLegend | 206D | 320114 |
| pS6 (S235/236) | AF488 | 1:50 | Cell Signaling Technology | D57.2.2E | 4803S |
| pAKT (S473) | PE-CF594 | 1:20 | BD Biosciences | M89-61 | 562465 |
| pAKT (T308) | PE | 1:20 | BD Biosciences | J1-223.371 | 558275 |
| pPDPK1/pPDK1 (S241) | AF647 | 1:20 | BD Biosciences | J66-653.44.17 | 560091 |
| pSTAT3 (Y705) | PE | 1:50 | BD Biosciences | 4/P-STAT3 | 612569 |
| pSTAT4 (Y693) | AF488 | 1:50 | BD Biosciences | 38/p-Stat4 | 558136 |
| pSTAT5 (Y694) | PerCP-Cy5.5 | 1:50 | BD Biosciences | 47/Stat5 (pY694) | 560118 |
| pSTAT6 (Y641) | AF647 | 1:50 | BD Biosciences | 18/p-Stat6 | 612601 |

### Supplemental Table S3. List of antibodies used to analyze intracellular cytokines.

| **Target** | **Fluorochromes** | **Dilution** | **Company** | **Clone** | **Reference** |
| --- | --- | --- | --- | --- | --- |
| CD4 | FITC | 1:20 | BD Biosciences | RPA-T4 | 555346 |
| Live/Dead | Near Infra-Red | 1:500 | Thermo Fisher Scientific | N.A. | L10119 |
| IFN-γ | PE-Cy7 | 1:50 | BD Biosciences | 4S.B3 | 560741 |
| TNF-α | BUV395 | 1:50 | BD Biosciences | MAb11 | 563996 |
| IL-2 | BV650 | 1:50 | BD Biosciences | 5344.111 | 563947 |
| IL-4 | BUV737 | 1:50 | BD Biosciences | MP4-25D2 | 612835 |
| IL-5 | PE | 1:50 | BD Biosciences | TRFK5 | 554395 |
| IL-17 | BV786 | 1:50 | BD Biosciences | N49-653 | 563745 |
